# Low high-density lipoprotein cholesterol levels predicting poor outcomes in patients with hepatitis B virus-related acute-on-chronic liver failure

**DOI:** 10.3389/fmed.2022.1001411

**Published:** 2022-11-25

**Authors:** Yue Zhang, Peng Chen, Yun Zhang, Yuan Nie, Xuan Zhu

**Affiliations:** Department of Gastroenterology, Jiangxi Clinical Research Center for Gastroenterology, The First Affiliated Hospital of Nanchang University, Nanchang, Jiangxi, China

**Keywords:** high-density lipoprotein cholesterol, hepatitis B virus, acute-on-chronic liver failure, prognosis, CTP score, MELD score, COSSH-ACLF II score

## Abstract

**Background:**

Lipid profile disorders frequently occur in patients with advanced liver diseases. High-density lipoprotein cholesterol (HDL-C) levels decrease rapidly during acute conditions of some diseases, and HDL-C levels may be related to mortality in patients with hepatitis B virus-related acute-on-chronic liver failure (HBV-ACLF).

**Materials and methods:**

A retrospective cohort study was conducted on 200 subjects with HBV-ACLF. The patients were separated into non-survivors and survivors according to their 28-day outcome. Univariate and multivariate Cox regression analyses were performed to identify predictors of mortality, and the performance of these predictors was evaluated by receiver operating characteristic (ROC) curve analysis. Kaplan–Meier analysis was performed to draw survival curves of HDL-C.

**Results:**

The 28-day mortality in the cohort was 27.0%. HDL-C levels differed markedly between non-survivors and survivors. In the multivariate analysis, HDL-C, the Child-Turcotte-Pugh (CTP), model for end-stage liver disease (MELD), and Chinese Group on the Study of Severe Hepatitis B-ACLF II (COSSH-ACLF II) scores were identified as independent predictors for mortality (HR = 0.806, 95% CI: 0.724–0.898; HR = 1.424, 95% CI: 1.143–1.775; HR = 1.006, 95% CI: 1.002–1.007; and HR = 1.609, 95% CI: 1.005–2.575, respectively). Patients with lower HDL-C levels had a worse prognosis than those with higher HDL-C levels. In ROC analysis, the prognostic accuracy for mortality was similar between HDL-C (AUROC: 0.733) and the CTP, MELD, and COSSH-ACLF II scores (AUROC: 0.753; 0.674 and 0.770, respectively).

**Conclusion:**

The HDL-C level may serve as a potential indicator for the prognosis of HBV-ACLF and can be used as a simple marker for risk assessment and selection of therapeutic options.

## Introduction

Chronic hepatitis B virus (HBV) infection is the leading cause of acute-on-chronic liver failure (ACLF), and there are currently 350 million individuals infected with HBV worldwide (1). ACLF is characterized by organ failure, acute deterioration of liver function and high short-term mortality in the presence of preexisting chronic liver diseases, which usually progress rapidly (2). The clinical management of ACLF currently focuses on the treatment of complications and multiorgan-supportive care. Therefore, to timely distinguish patients with a high mortality risk from patients with a low mortality risk is helpful for improving the survival of HBV-ACLF.

Numerous risk score models have been reported to predict poor outcomes in patients with ACLF, such as the Child-Turcotte-Pugh (CTP) score, Model for End-Stage Liver Disease (MELD) score, and Chinese Group on the Study of Severe Hepatitis B-ACLF (COSSH-ACLF) II score (3–5). However, there are some limitations between these score models. The CTP score consists of two subjective parameters, hepatic encephalopathy and ascites, which are prone to deviate between different evaluators (6). The MELD score is calculated by serum creatinine, bilirubin, and INR, while its deficiency lies in the intricacy of its calculation (6, 7). The COSSH-ACLF II score was established based on a large cohort of HBV-ACLF patients and has a high prognostic value; however, the COSSH-ACLF II score includes subjective indices such as hepatic encephalopathy, and the calculation is very complex (5). Therefore, the identification of certain objective indicators obtained from routine blood tests that can provide a predictive value for patients with HBV-ACLF is warranted.

The liver is an indispensable organ for several stages of lipoprotein and lipid synthesis, metabolism, and secretion (8). Alterations in serum lipid levels are common in cirrhotic patients, especially changes in serum high-density lipoprotein cholesterol (HDL-C) (9). Moreover, low HDL-C levels are significantly associated with mortality in patients with cirrhosis (10), which is also related to infectious-related mortality (11). He et al. (12) identified HDL-C as an independent predictor for poor short-term outcomes in patients with HBV-associated decompensated cirrhosis. Recently, Wen et al. (13) demonstrated that HDL-C had good prognostic value in predicting 1-year survival, but it did not do well in predicting the 90-day outcome of HBV-ACLF patients. In light of these findings, we investigated whether serum HDL-C levels can predict short-term outcomes in HBV-ACLF patients.

## Materials and methods

### Patients

A total of 200 patients diagnosed with HBV-ACLF were retrospectively recruited from May 2018 to May 2021 at the Department of Gastroenterology, First Affiliated Hospital of Nanchang University. The inclusion criteria were as follows: (1) age ≥18 years; (2) positive tests for HBV DNA and hepatitis B surface antigen (HBsAg) for >6 months; and (3) ACLF diagnosed based on the diagnostic criteria recommended by the Asian Pacific Association for the Study of the Liver (APASL). The exclusion criteria were as follows: (1) combined with hepatocellular carcinoma (HCC); (2) infection with human immunodeficiency virus; (3) coinfection with hepatitis A/C/D/E virus; and (4) incomplete data (5) complicated with other severe chronic extrahepatic disease. The study was approved by the Ethics Committee of the First Affiliated Hospital of Nanchang University.

### Data collection and follow-up

The following demographic and clinical data were collected from the patients’ medical records. Laboratory parameters were measured in all subjects using fasting venous blood samples in the first 24-h period during hospitalization. All HBV-ACLF patients were followed up for 28 days, and the 28-day mortality rate was determined. The calculation methods of the CTP and MELD scoring systems were previously described (14, 15). The COSSH-ACLF II scores were calculated using the formula: 1.649 × ln(INR) + 0.457 × HE score (HE grade: 0/1, 1–2/2 and 3–4/3) + 0.425 × ln(neutrophil) (10^9^/L) + 0.396 × ln(TB) (μmol/L) + 0.576 × ln(serum urea) (mmol/L) + 0.033 × age (5).

### Definitions

According to the APASL criteria proposed in 2019, patients were diagnosed with ACLF when they meet the following conditions: (1) preexisting chronic liver diseases (diagnosed or undiagnosed); (2) coagulopathy (international normalized ratio [INR] ≥ 1.5 or prothrombin activity <40%); (3) jaundice (serum total bilirubin ≥ 5 mg/dL); (4) complicated with ascites or hepatic encephalopathy within 4 weeks; and (5) a high 28-day mortality (16). HBV-ACLF was diagnosed as HBsAg-positive ≥6 months complicated with ACLF.

### Statistical analysis

Data analysis was performed by Statistical Product and Service Solutions (SPSS) software version 24.0 (SPSS Inc., Chicago, IL, United States) and R software version 4.1.0 (The R Foundation for Statistical Computing),^1^ and ROC analysis was performed by using MedCalc statistical software version 15.2.1 (MedCalc, Ostend, Belgium). Continuous variables were expressed as the means ± standard deviation (SD) if data were normally distributed and as the medians and interquartile range if data were skewed. Categorical variables are expressed as frequencies and proportions and were compared by the chi-square test or Fisher’s exact test. Normally distributed continuous variables were compared by independent Student’s *t*-test, and skewed continuous variables were compared by the Mann–Whitney *U*-test. Univariate and multivariate Cox regression analyses were performed to evaluate the relationships between clinical variables and prognosis in HBV-ACLF patients. Univariate risk factors that reached *p* < 0.10 were subjected to multivariate Cox regression analysis. The survival curves were calculated using the “survival,” “rms,” and “survminer” packages in R software, of which the ggsurvplot function was used to generate the K-M survival curve. Receiver operating characteristic (ROC) curves were used to measure the performance of HDL-C and scores for the prediction of 28-day mortality in HBV-ACLF patients. The Delong test was used to compare the AUROC of HDL-C and scores. A two-sided *p*-value < 0.05 was considered significantly different.

## Results

### Characteristics of hepatitis B virus-related acute-on-chronic liver failure patients

**FIGURE 1:**
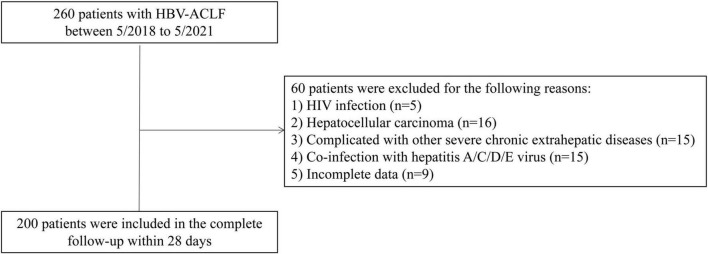
Flow chart for hepatitis B virus-related acute-on-chronic liver failure (HBV-ACLF) patients in the study.

A total of 200 HBV-ACLF patients were included in this retrospective study (Figure 1). The comparison of the demographic and clinical characteristics of HBV-ACLF patients stratified by mortality is presented in Table 1. A total of 54 (27.0%) patients died within 28 days after admission. The mean ages of the entire group, survivors and non-survivors were 48.25 ± 11.69, 46.99 ± 11.21, and 51.67 ± 12.36, respectively. Male patients accounted for 87.0% of the overall population. There were no significant differences in sex distribution, hospitalization expense, white blood cell count (WBC), Hb, alanine aminotransferase (ALT), or aspartate aminotransferase (AST) between the non-survivor group and the survivor group (*P* > 0.05). However, marked differences were observed between the survivors and non-survivors in age, platelet (PLT), INR, prothrombin time (PT), albumin, creatinine, blood urea nitrogen, bilirubin, total cholesterol, CTP score, MELD score, COSSH-ACLF II score, and HDL-C level (all *P* < 0.05).

**TABLE 1:** Baseline characteristics of HBV-ACLF patients.

	All patients (*n* = 200)	Surviving patients (*n* = 146)	Non-surviving patients (*n* = 54)	*P*
Male [n (%)]	174 (87.0)	127 (86.9)	47 (87.0)	0.824
Age (years)	48.25 ± 11.69	46.99 ± 11.21	51.67 ± 12.36	**0.012**
Expense (USD)	10922.65 (6759.39–17669.28)	11334.59 (6838.99–17459.69)	9705.85 (6255.59–18040.91)	0.595
WBC (10^9^/L)	6.590 (5.158–8.368)	6.550 (5.202–8.238)	6.695 (4.820–9.015)	0.453
PLT (10^9^/L)	110.50 (79.00–140.75)	115.00 (86.00–145.00)	86.50 (51.00–134.50)	**0.008**
INR	1.96 (1.70–2.52)	1.90 (1.69–2.36)	2.09 (1.76–2.84)	**0.018**
PT (s)	21.95 (19.20–27.93)	21.15 (18.98–25.73)	23.80 (19.98–31.48)	**0.019**
Hb (g/L)	124.50 (110.00–137.75)	125.00 (113.00–140.00)	121.50 (103.75–133.75)	0.134
Albumin (g/L)	31.47 ± 4.52	32.08 ± 4.55	29.81 ± 4.01	**0.001**
Creatinine (μmol/L)	66.45 (57.35–81.15)	64.70 (57.08–78.25)	72.65 (58.63–92.60)	**0.048**
BUN (mmol/L)	4.00 (2.70–5.58)	3.75 (2.60–5.20)	5.15 (3.32–5.52)	**0.001**
ALT (μ/L)	364.00 (127.50–887.00)	291.45 (128.70–716.13)	409.50 (125.50–950.75)	0.373
AST (μ/L)	245.50 (131.55–581.75)	232.00 (123.75–608.75)	263.15 (162.25–554.00)	0.391
Bilirubin (μmol/L)	315.90 ± 135.73	302.49 ± 123.30	352.15 ± 160.47	**0.021**
TC (mg/dL)	97.81 (78.19–118.69)	104.58 (80.70–124.00)	84.28 (69.59–101.68)	**<0.001**
HDL-C (mg/dL)	8.12 (5.90–10.82)	8.51 (6.57–12.47)	6.57 (4.64–8.51)	**<0.001**
CTPs	11.00 (10.00–12.00)	11.00 (10.00–12.00)	12.00 (11.00–13.00)	**<0.001**
MELDs	22.59 (19.57–26.36)	21.61 (18.85–25.49)	24.45 (21.73–28.50)	**<0.001**
COSSH-ACLF IIs	6.97 (6.41–7.77)	6.73 (6.30–7.25)	7.72 (6.99–8.65)	**<0.001**

*P*-value < 0.05 was considered significant and was indicated in bold. ACLF, acute on chronic liver failure; HBV, hepatitis B Virus; USD, dollar; WBC, white blood cell count; PLT, platelet; INR, international normalized ratio; PT, prothrombin time; Hb, hemoglobin; BUN, blood urea nitrogen; ALT, alanine aminotransferase; AST, aspartate aminotransferase; TC, total cholesterol; HDL-C, high-density lipoprotein cholesterol; CTPs, child-turcotte-pugh score; MELDs, model for end-stage liver disease score; COSSH-ACLF IIs, Chinese Group on the Study of Severe Hepatitis B-ACLF II score.

### Factors related to mortality

As shown in Table 2, clinical and laboratory data were investigated for the prediction of mortality by univariate Cox regression analyses. In the univariate analyses, PLT, total cholesterol, HDL-C, and CTP, MELD, and COSSH-ACLF II scores were associated with mortality (all *P* < 0.05). Parameters with *P*-values were less than 0.1 in univariate Cox regression were included in multivariate Cox regression analysis, which showed that HDL-C, CTP score, MELD score, and COSSH-ACLF II score were independent predictors of mortality (HR = 0.806, 95% CI: 0.724–0.898; HR = 1.424, 95% CI: 1.143–1.775; HR = 1.006, 95% CI: 1.002–1.007; and HR = 1.609, 95% CI: 1.005–2.575, respectively).

**TABLE 2:** Univariate and multivariate analyses of risk factors associated with mortality in hepatitis B virus-related acute-on-chronic liver failure (HBV-ACLF) patients.

	Univariate			Multivariate		
	HR	95% CI	*P*-value	HR	95% CI	*P*-value
**Sex**						
Female	Reference					
Male	1.057	0.478–2.339	0.891			
WBC (10^9^/L)	1.059	0.999–1.122	0.053			
PLT (10^9^/L)	0.993	0.988–0.999	**0.013**			
ALT (μ/L)	1.001	0.999–1.002	0.397			
AST (μ/L)	1.002	0.998–1.003	0.145			
TC (mg/dL)	0.983	0.975–0.992	**<0.001**			
HDL-C (mg/dL)	0.811	0.730–0.900	**<0.001**	0.806	0.724–0.898	**<0.001**
CTPs	1.685	1.418–2.002	**<0.001**	1.424	1.143–1.775	**0.002**
MELDs	1.158	1.022–1.196	**0.002**	1.006	1.002–1.007	**0.048**
COSSH-ACLF IIs	1.886	1.532–2.321	**<0.001**	1.609	1.005–2.575	**0.025**

*P*-value < 0.05 was considered significant and was indicated in bold. WBC, white blood cell count; PLT, platelet; ALT, alanine aminotransferase; AST, aspartate aminotransferase; TC, total cholesterol; HDL-C, high-density lipoprotein cholesterol; CTPs, child-turcotte-pugh score; MELDs, model for end-stage liver disease score; COSSH-ACLF IIs, Chinese Group on the Study of Severe Hepatitis B-ACLF II score.

### Low expression of high-density lipoprotein cholesterol predicts poor prognosis of hepatitis B virus-related acute-on-chronic liver failure

**FIGURE 2:**
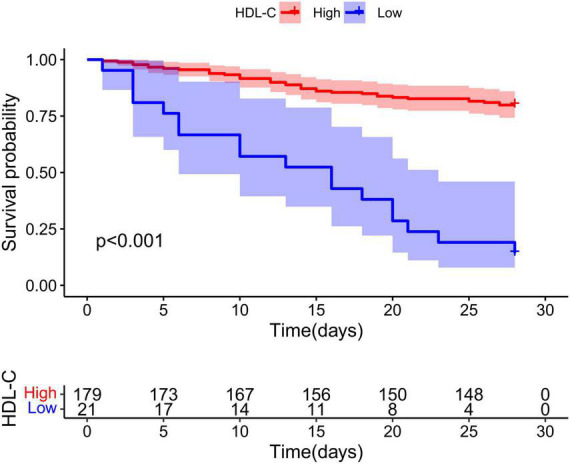
Low high-density lipoprotein cholesterol (HDL-C) expression predicts poor prognosis of hepatitis B virus-related acute-on-chronic liver failure (HBV-ACLF). In HBV-ACLF patients, the cumulative survival rates of the HDL-C low expression group and high expression group were analyzed by Kaplan–Meier curve, and the difference in cumulative survival rates between groups was determined by the log rank test.

As shown in Figure 2, Kaplan–Meier analysis was performed to evaluate HDL-C in HBV-ACLF patients, and patients with low HDL-C expression had a poorer prognosis than those with high HDL-C expression (*P* < 0.001). The cutoff value of HDL-C was 4.64 according to the data analysis.

### Comparison of clinical features between patients with low and high high-density lipoprotein cholesterol levels

The HBV-ACLF patients were stratified into two groups based on the cutoff value for baseline HDL-C (≤4.64 mg/dL, *n* = 21 vs. >4.64 mg/dL, *n* = 179). Patients with HDL-C ≤ 4.64 mg/dL had lower platelets, older ages, higher WBCs, higher blood urea nitrogen, higher bilirubin, lower total cholesterol, higher CTP scores, higher MELD scores, higher COSSH-ACLF II scores, and higher mortality (Table 3).

**TABLE 3:** Clinical data according to HDL-C levels.

	Low HDL-C group (*n* = 21)	High HDL-C group (*n* = 179)	*P*
Male [n (%)]	18 (85.7)	156 (87.2)	1.000
Age (years)	57.00 ± 13.40	47.22 ± 11.07	**<0.001**
Expense (USD)	9479.7 (5448.1–17680.8)	11238.2 (6761.9–17756.1)	0.482
WBC (10^9^/L)	6.67 (5.25–8.53)	5.53 (4.40–7.50)	**0.045**
PLT (10^9^/L)	81.00 (48.00–121.50)	114.00 (82.00–144.00)	**0.012**
INR	2.12 (1.64–2.68)	1.94 (1.71–2.52)	0.634
PT (s)	23.60 (18.45–28.45)	21.80 (19.20–27.85)	0.785
Hb (g/L)	126.00 (108.00–139.00)	123.00 (110.00–138.00)	0.757
Albumin (g/L)	30.92 ± 2.78	31.53 ± 4.68	0.558
Creatinine (μmol/L)	74.20 (60.80–97.05)	64.90 (57.20–79.80)	0.110
BUN (mmol/L)	5.40 (3.60–8.35)	4.00 (2.60–5.45)	**0.007**
ALT (μ/L)	383.00 (126.00–947.00)	253.00 (111.35–625.35)	0.291
AST (μ/L)	198.00 (142.50–351.25)	249.00 (131.40–629.00)	0.511
Bilirubin (μmol/L)	382.77 ± 137.96	308.05 ± 133.68	**0.017**
TC (mg/dL)	85.43 (68.23–102.06)	100.90 (78.48–121.01)	**0.018**
CTPs	12.00 (10.50–12.00)	11.00 (10.00–12.00)	**0.046**
MELDs	25.04 (22.01–28.72)	22.21 (19.55–26.23)	**0.021**
COSSH-ACLF IIs	7.63 (7.08–8.64)	6.88 (6.36–7.69)	**0.001**
28-days mortality [n (%)]	18 (85.71)	36 (20.11)	**<0.001**

*P*-value < 0.05 was considered significant and was indicated in bold. ACLF, acute on chronic liver failure; HBV, hepatitis B Virus; USD, dollar; WBC, white blood cell count; PLT, platelet; INR, international normalized ratio; PT, prothrombin time; Hb, hemoglobin; BUN, blood urea nitrogen; ALT, alanine aminotransferase; AST, aspartate aminotransferase; TC, total cholesterol; HDL-C, high-density lipoprotein cholesterol; CTPs, child-turcotte-pugh score; MELDs, model for end-stage liver disease score; COSSH-ACLF IIs, Chinese Group on the Study of Severe Hepatitis B-ACLF II score.

### Predictive value of high-density lipoprotein cholesterol in hepatitis B virus-related acute-on-chronic liver failure patients

**FIGURE 3:**
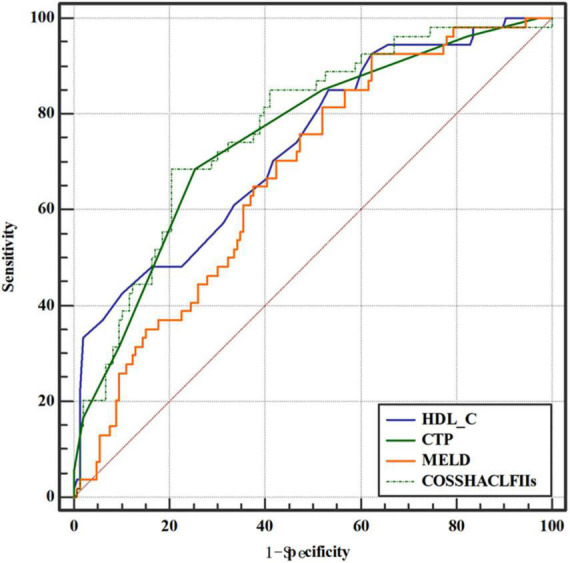
Area under the receiver operating characteristic (ROC) curve comparison of high-density lipoprotein cholesterol (HDL-C) and the child-turcotte-pugh (CTP), model for end-stage liver disease (MELD), and Chinese Group on the Study of Severe Hepatitis B-ACLF II (COSSH-ACLF II) scores for the prediction of poor outcomes in hepatitis B virus-related acute-on-chronic liver failure (HBV-ACLF) patients.

To investigate the relationship between HDL-C level and prognosis in patients with HBV-ACLF, we conducted area under the receiver operating characteristic (ROC) curve analysis (AUROC) to compare the prognostic value of HDL-C level and other score models. At 28 days, the serum HDL-C level exhibited strong prognostic ability, with a high AUROC value [0.733 (95% CI: 0.666–0.793)], which was comparable to the AUROC values obtained with the COSSH-ACLF II score [0.770 (95% CI: 0.705–0.826)], CTP score [0.753 (95% CI: 0.688–0.811)], and MELD score [0.674 (95% CI: 0.604–0.738)] (*P* > 0.05 for all comparisons; Tables 4, 5). The prognostic accuracies of HDL-C and score models for the prediction of mortality according to ROC curves are shown in Figure 3.

**TABLE 4:** The performance of high-density lipoprotein cholesterol (HDL-C) and the prognostic scores for predicting outcome at 28 days.

Prognostic score	ROC area (95%CI)	*P*-value
**28-day mortality**		
HDL-C	0.733 (0.666–0.793)	**<0.001**
CTP	0.753 (0.688–0.811)	**<0.001**
MELD	0.674 (0.604–0.738)	**<0.001**
COSSH-ACLF IIs	0.770 (0.705–0.826)	**<0.001**

*P*-value < 0.05 was considered significant and was indicated in bold. CTP, Child-Turcotte-Pugh; MELD, model for end-stage liver disease; COSSH-ACLF IIs, Chinese Group on the Study of Severe Hepatitis B-ACLF II score.

**TABLE 5:** The comparison of predictive value between high-density lipoprotein cholesterol (HDL-C) and scores.

Prognostic score	Difference between areas (95%CI)	*P*-value
**28-day mortality**		
HDL-C vs. CTPs	0.0204 (−0.0996 to 0.1400)	0.739
HDL-C vs. MELDs	0.0594 (−0.0556 to 0.1740)	0.311
HDL-C vs. COSSH-ACLF IIs	0.0370 (−0.0705 to 0.1450)	0.500

CTP, Child-Turcotte-Pugh; MELD, model for end-stage liver disease; COSSH-ACLF IIs, Chinese Group on the Study of Severe Hepatitis B-ACLF II score.

## Discussion

In the present study, we show that HDL-C levels are robust predictors of 28-day mortality in HBV-ACLF patients. The prognostic values of HDL-C were very similar to those of the COSSH-ACLF II, CTP, and MELD scores.

Several common scores have been confirmed as predictors of mortality in patients with HBV-ACLF. The Child–Pugh score was originally used to predict outcome in patients with cirrhosis undergoing surgery (14). The MELD score was initially established to predict the outcome of patients who underwent transjugular intrahepatic portosystemic shunt insertions (15). The COSSH-ACLF II score was first developed by the HE score, INR, total bilirubin, neutrophils, serum urea, and age and has been confirmed to have better predictive value than other scores in predicting the short-term mortality of HBV-ACLF patients (5). In our study, we found that non-survivors had lower HDL-C levels than survivors. Moreover, decreased HDL-C was independently associated with 28-day mortality in HBV-ACLF patients, with a predictive power similar to that of the CTP, MELD, and COSSH-ACLF II scores. Among them, HDL-C was the most readily available indicator. HDL-C can be easily measured in routine clinical laboratories and shows good prognostic value for short-term mortality in HBV-ACLF. Notably, Trieb et al. (17) included 548 patients and divided them into four groups, including the control, stable cirrhosis, acute decompensation and ACLF groups. The author found that HDL-C was significantly lower in patients with ACLF than in controls and patients with stable cirrhosis. Moreover, HDL-C showed high predictive value for the outcome of 90-day mortality, which was similar to that of the MELD and CLIF-C ACLF scores. Moreover, a previous study confirmed that HDL-C exhibited certain prognostic potential in patients with HBV-ACLF (13). Our study complements these studies and demonstrates that HDL-C can be utilized to predict short-term prognosis in HBV-ACLF patients.

The potential mechanism linking HDL-C and prognosis in HBV-ACLF patients should be considered. We found that HDL-C was markedly lower in the non-survivors than in the survivors. Moreover, WBC was significantly higher in the low HDL-C group than in the high HDL-C group. As a single precipitating event, HBV may induce the occurrence of ACLF; furthermore, systemic inflammation may contribute to the pathogenesis of ACLF (18). A previous study showed that cirrhosis obviously damaged the ability of HDL to suppress NF-kB activation and cytokine production (19). Furthermore, researchers have demonstrated that HDL-C generally plays an important anti-inflammatory role by binding and neutralizing bacterial lipopolysaccharides (LPS) (20, 21). There is clear evidence confirming that inflammation significantly changes the structure and function of HDL, which may cause the proinflammatory forms of HDL (22). An *in vivo* study in patients with advanced chronic liver failure confirmed that the proinflammatory cytokines produced by LPS were abolished by HDL incubation (23). Accordingly, the decrease in HDL may induce a reduction in anti-inflammatory activity, which may contribute to the poor prognosis in HBV-ACLF patients.

The limitations of our study should be recognized. First, the single-center and retrospective nature of the study may have led to selection bias. Second, the HDL-C level was not dynamically detected during follow-up. Finally, this study only included Asian patients, so the conclusions may not apply to Europeans.

In conclusion, our study indicates that HDL-C is an independent predictor of 28-day mortality in HBV-ACLF patients. The prognostic value of HDL-C as a single biomarker was similar to that of the CTP, MELD, and COSSH-ACLF II scores. HDL-C may be a simple and accurate prognostic marker for short-term mortality in HBV-ACLF patients.

## Data availability statement

The raw data supporting the conclusions of this article will be made available by the authors, without undue reservation.

## Ethics statement

The studies involving human participants were reviewed and approved by the Ethics Committee of First Affiliated Hospital of Nanchang University. The patients/participants provided their written informed consent to participate in this study.

## Author contributions

YueZ designed and wrote the original draft. PC analyzed the data and wrote the original draft. YunZ collected the data and wrote the original draft. YN and XZ critically revised the manuscript. All authors contributed to the article and approved the submitted version.

## Footnotes

1
https://www.r-project.org/
